# BioControl 3.0: Biological Control Complex for Pest Control—Enhanced Control of *Locusta migratoria manilensis* via Combined Application of *Metarhizium anisopliae* var. *acridum* and *Carabus smaragdinus*

**DOI:** 10.3390/ani16020345

**Published:** 2026-01-22

**Authors:** Linqiang Gao, Yan Wang, Ruxin Wang, Jinshu Yang, Meiyi Yang, Yusheng Liu, Guangjun Wang, Mark R. McNeill, Zehua Zhang, Xinghu Qin, Haiyan Wang

**Affiliations:** 1 School of Ecology and Nature Conservation, Beijing Forestry University, Beijing 100087, China; 18734246491@163.com (L.G.); wy3240742@bjfu.edu.cn (Y.W.);; 2 The International Machine Learning Laboratory for Biodiversity and One Health Research, Beijing Forestry University, Beijing 100083, China; 3 School of Information Science and Technology, Beijing Forestry University, Beijing 100083, China; 4 Agricultural Technology Extension Center, Qiandongnan Prefecture, Kaili 556000, China; 5 School of Forestry, Beijing Forestry University, Beijing 100087, China; 6 College of Forestry Engineering, Shandong Agriculture and Engineering University, Jinan 250100, China; 7 Institute of Plant Protection, Chinese Academy of Agricultural Sciences, Beijing 100081, China; 8 Biocontrol & Biosecurity, AgResearch, Private Bag 4749, Christchurch 8140, New Zealand

**Keywords:** additive effect, biological control, locusts, predator-pathogen complex

## Abstract

*Locusta migratoria manilensis* (Meyen) is a highly destructive insect pest worldwide. However, excessive reliance on insecticides has resulted in significant environmental pollution. Biocontrol complexes combine two or more BCAs to address the limitations of individual agents. However, biocontrol complex for locust control has been rarely reported. Here, we propose BioControl 3.0, which integrates *Metarhizium anisopliae* var. *acridum* (Driver and Milner) and *Carabus smaragdinus* (Fischer von Waldheim) for locust control. We evaluated this system through a series of laboratory bioassays and semi-field cage experiments, comparing single-agent applications, sequential combinations (BioControl 2.0), and predator-mediated delivery (BioControl 3.0), and quantified locust mortality and interaction effects between predation and infection We found that *M. anisopliae* caused >85% mortality of locust nymphs at 1 × 10^8^ conidia/mL (LT_50_ ≈ 6 days) while exhibiting negligible virulence toward *C. smaragdinus.* BioControl 2.0 (sequential application) increased mortality compared to single agents. However, this approach revealed a significant negative interaction between predation and infection, which limited the total control efficacy. BioControl 3.0 (predator-vectored fungus) achieved the highest corrected mortality, with predation and infection acting independently and additively (no detectable antagonistic interaction). By leveraging a predatory vector, BioControl 3.0 decouples negative interaction and harnesses dual biotic pressures, offering a cost-effective, environmentally benign alternative to conventional locust control. Our findings provide a blueprint for designing integrated predator-pathogen complexes and optimizing deployment strategies for sustainable management of locust outbreaks.

## Simple Summary

The migratory locust is a major agricultural pest. While chemical control is common, it poses significant environmental and health risks. This study proposes “BioControl 3.0,” a novel strategy that combines the insect-killing fungus *Metarhizium anisopliae* with the predatory beetle *Carabus smaragdinus*. BioControl 3.0, the predator-vectored delivery complex, achieved notable success rate when controling locusts, because the beetle and fungus worked independently and additively. The fungus was also found to be safe for the beetles and can even help promote plant growth in previous studies. This integrated approach provides a more effective, environmentally friendly, and sustainable blueprint for managing locust outbreaks.

## 1. Introduction

Insecticides have long been the mainstay for controlling pests such as the migratory locust (*Locusta migratoria manilensis* Meyen). However, their overuse has not only posed a threat to humans and wildlife but also led to pest resistance, recurrent outbreaks, and severe environmental contamination [1,2,3]. Consequently, biological control, the use of living organisms or their natural products to suppress pests, has attracted attention as an environmentally friendly alternative. This approach employs beneficial organisms such as fungi, bacteria, insect viruses, and natural enemies, as well as their byproducts, to target specific plant pests, diseases, and weeds. Compared to conventional insecticides, biological control offers advantages including reduced environmental toxicity, absence of chemical residues, and enhanced compatibility with human safety [4,5]. Nevertheless, widespread adoption of biocontrol agents (BCAs) remains constrained by inconsistent field efficacy, economic viability challenges, and insufficient empirical validation of long-term benefits [6,7,8].

*L. migratoria* is a globally destructive pest, which has caused significant agricultural losses in the past [9]. Various approaches have been proposed to manage locust infestations, including insecticides, biopesticides, biological agents, and physical management methods like burning roosting locusts or digging trenches to bury them [10,11,12,13]. These methods can rapidly reduce locust densities, but chemical control remains the primary response to outbreaks, despite well-documented drawbacks such as non-target impacts and the evolution of insecticide resistance [14,15]. Consequently, there is a growing interest in biological control options, either as standalone measures or as part of integrated pest management (IPM) strategies [16,17].

Biological control is inherently a biological and ecological process, as it relies on natural interactions such as predation, parasitism, and pathogen transmission to regulate pest populations. The success of biocontrol agents is therefore strongly influenced by ecological compatibility, including spatial and temporal overlap between agents and target pests, behavioral interactions, and environmental persistence. Moreover, when multiple biocontrol agents are combined, their interactions may be additive, synergistic, or antagonistic, depending on how they interact within the ecological system. Understanding these biological and ecological dimensions is essential for designing effective and sustainable biocontrol strategies, particularly in multi-agent systems.

A wide variety of BCAs have been investigated against locusts. Notable examples include the microbes like *Metarhizium anisopliae* [18], *Nosema locustae* [19], *Pseudomonas pseudoaligenes* [20] and *Bacillus thuringiensis* [5]; entomopoxviruses [21]; predatory beetles like Cantharid [22], *Calosoma maximoviczi* Mora [23], and *Carabus smaragdinus* Fischer (Coleoptera, Carabidae) [24]; parasitoids such as *Anastoechus* spp., *Blaesoxipha* (Blaesoxipha) *migratoriae* Rohdendorf [17,25,26,27]; pheromones [28]; and avian species [29]. These agents form the basis of traditional “BioControl 1.0” approaches.

Among these, *Metarhizium anisopliae* stands out as a highly effective entomopathogenic fungus used to control various agricultural pests with minimal harm to livestock and humans [30]. Commercial formulations of *M. anisopliae* have been deployed against over 200 arthropod pests, demonstrating substantial economic and ecological benefits [31]. *Metarhizium anisopliae* var. *acridum*, a specific fungal isolate within the genus, is known for its virulence against grasshoppers, including *L. migratoria* [17]. This fungal strain has demonstrated an average field mortality rate of 85% in locust populations while having minimal negative effects on non-target organisms [17].

Predatory beetles, particularly *Carabus smaragdinus* (Coleoptera: Carabidae), represent a complementary control strategy. Native to North China, *C. smaragdinus* is a voracious ground predator that readily consumes locust nymphs and various lepidopteran larvae [24,32]. Observations indicate that this species exhibits rapid hunting behavior and strong prey responsiveness, positioning it as a promising candidate for augmenting biocontrol efforts.

Despite various types of single BCAs, adoption remains limited due to variable field performance and concerns over cost-effectiveness [1,7,8]. To enhance control efficacy, researchers have begun combining BCAs into synergistic complexes (“BioControl 2.0”) [5,33]. The biocontrol complex could address the limitations of single-agent approaches, potentially boosting efficacy through complementary actions. For example, a combination of *M. anisopliae* and the bacterium *Pseudomonas pseudoaligenes* has been shown to exert a greater suppressive effect on locust populations than either agent alone [17]. Building on previous work on single-agent and combined biological control strategies, the objective of this study was to evaluate a novel predator-pathogen complex for locust management, referred to here as BioControl 3.0. Specifically, we aimed to test whether using a predatory beetle as a biological vector for an entomopathogenic fungus could overcome the limitations commonly observed in conventional biocontrol systems, including inconsistent efficacy and negative interactions between agents. Using *Locusta migratoria manilensis* as a model pest, we investigated a system in which adult *Carabus smaragdinus* were pre-inoculated with *Metarhizium anisopliae* var. *acridum* prior to release. The study was designed to (i) assess the virulence and biosafety of *M. anisopliae* toward both the target pest and the predator, (ii) compare the effectiveness of single-agent, sequential, and vector-mediated biocontrol strategies under semi-field conditions, and (iii) analyze the interaction dynamics between predation and fungal infection under different deployment configurations. By addressing these objectives, this work seeks to provide an empirical framework for optimizing integrated biological control systems for sustainable locust management.

## 2. Materials and Methods

### 2.1. Source of L. migratoria, C. smaragdinus, and M. anisopliae

Locusts (*L. migratoria*) were obtained from rearing facilities at the Experimental Station of the College of Plant Protection, Shandong Agricultural University (ES-CPP, SDAU) in Taian. The locusts were reared on wheat seedlings grown in a glasshouse. *Carabus smaragdinus* adults were sourced from the ES-CPP, SDAU workshop and were reared on larvae of the mealworm (*Tenebrio molitor* L.).

The strain of *M. anisopliae* used in these experiments was MA2013107391001A, which is identified from field collections in 2015 and preserved at Shandong Agricultural University. It is registered for use against *L. migratoria* under patent ZL201310739100.1 [32].

### 2.2. Experimental Design

Three experiments were conducted to evaluate the potential of both the entomopathogen and the predator on *L. migratoria*. Experiment 1 (virulence assay) quantified the pathogenicity of *M. anisopliae* against locusts. Experiment 2 (biosafety assay) assessed the effect of then fungus on adult *C. smaragdinus* survival. Experiment 3 (field-cage trials) comprised several separate experiments designed to evaluate the combined efficacy of the beetle–fungus system in outdoor cages. Specifically, Experiment 3a examined the performance of *M. anisopliae* alone under field-cage conditions, Experiment 3b assessed the predatory capacity of *C. smaragdinus* alone, Experiment 3c tested BioControl 2.0 (sequential application of fungus and predator), and Experiment 3d evaluated BioControl 3.0 (predator-vectored fungus). In all experiments, third-instar locusts were used, and the female-to-male ratio was maintained at 1:1. Recently emerged adult beetles (6–8 days old) of *C. smaragdinus* were collected for the experiments.

### 2.3. Virulence Assay on Locusts

*M. anisopliae* was cultured on potato sucrose agar medium (PSA) at pH 7 and a temperature of 25 °C for seven days in a constant temperature incubator (GXZ-9240A) with a light–dark cycle of 12 h each. After the incubation period, the culture was harvested. Three independent 0.5 g samples of the product were taken and added to 10 mL of sterile deionized water containing 0.2% surfactant (Tween 80). This suspension was sonicated for 3 min, and the number of conidia was counted using a hemocytometer. The suspension was then diluted and standardized to final concentrations of 10^5^, 10^6^, 10^7^, and 10^8^ conidia/mL using 10 mL of sterile deionized water with 0.2% surfactant (Tween 80), with vortexing for 30 s at each dilution. Viability assessments were conducted by directly counting viable and non-viable conidia after 10–20 h under a light microscope at a magnification of 400×, focusing on a field in the center of each quadrant without altering the field of view. More than 200 conidia per plate on average were included in the analysis, and viable conidia were identified as those with germ tubes longer than their diameters [34]. A solution of 10 mL of sterile deionized water with 0.2% surfactant (Tween 80) was used as the control.

All treatments were prepared to a total volume of 10 mL. For each treatment, 2 μL was collected with a 2 μL pipette and evenly spread on the pronotum of each locust. Fifteen third-instar nymphs were selected and placed into a plastic container (30 cm × 20 cm × 15 cm) after inoculation. Each treatment of *M. anisopliae*, including the control, was replicated five times. The locusts were fed with 50 g of wheat seedlings and kept at a temperature of 28 °C and a humidity of 60–80% in an artificial climatic chamber (MCL-PQX, 250L, MCL, LTD., Guangzhou, China) with a light–dark cycle of 12 h each. Uneaten vegetation was replaced daily with fresh 50 g seedlings, and the number of dead individuals was recorded. Dead locusts were placed on filter paper in sterile Petri dishes (10 cm × 2.5 cm deep), with one cadaver per dish. To prevent desiccation, the filter paper was moistened with distilled water, and a cotton ball soaked in sterile water was placed in the center of the dish. Vegetation replacement and mortality assessment were performed between 08:00 and 20:00. The dishes were incubated at 30 °C and a light–dark cycle of 12 h each, and checked after 3–5 days to determine the incidence of infection based on the presence of muscardine on the cadavers.

### 2.4. Biosafety Assessment of M. anisopliae on C. smaragdinus

To evaluate the virulence of *M. anisopliae* on *C. smaragdinus*, we treated early adult beetles (6–8 days old) with a solution containing 2 μL of 10^8^ conidia/mL of *M. anisopliae*, along with 0.2% Tween 80. As a control, another group of beetles was treated with a 0.2% Tween 80 solution only. The treatments were evenly applied to the adult bodies of *C. smaragdinus* using a 2 μL plastic pipette. The beetles were reared in plastic containers measuring 1.0 m × 0.7 m × 0.5 m, containing approximately 5 cm of clean soil at the bottom. Owing to limited beetle availability, treatments were replicated three times, with each replicate containing 50 adult beetles at a 1:1 female-to-male ratio. The containers were placed in a temperature-controlled chamber at a constant temperature of 28 °C, with a humidity range of 60% to 80% and a light–dark cycle of 12 h each. To serve as food for the beetles, approximately 100 *T. molitor* larvae were provided daily to each container. Dead *C. smaragdinus* beetles were collected every three days between 06:00 and 22:00 and placed individually on sterile filter paper in Petri dishes measuring 10 cm × 2.5 cm deep. To prevent desiccation, the filter paper was moistened with distilled water, and a cotton ball soaked in sterile water was placed in the center of each dish. The Petri dishes were kept at a constant temperature of 30 °C with a light–dark cycle of 12 h each. Mortality was recorded daily for a period of 40 days.

### 2.5. Combined Efficacy Evaluation in Field Cages

The field experiment was conducted to evaluate the performance of the combination of *C. smaragdinus* and *M. anisopliae*. The cages constructed from mesh (mesh size: 1 mm), measuring 1 m × 1 m × 1 m, were placed over an area where all vegetation and surface arthropods were carefully removed, and the top 10 cm of soil was excavated to eliminate any insect eggs before introducing *C. smaragdinus* and *L. migratoria* into the cages. Five treatments were established in field cages. Each cage was positioned approximately 2 m apart, and the treatments were randomly assigned to the cages. Below is the setup of five treatments.

- Treatment I (BioControl 1.0 predator): 10 locusts + 2 untreated *C. smaragdinus* (1 female + 1 male).
- Treatment II (BioControl 1.0 fungus): 10 locusts treated with *M. anisopliae* (2 μL of a conidial suspension containing 1 × 10^8^ conidia per milliliter (conidia/mL)) + no beetles.
- Treatment III (BioControl 2.0): 10 locusts treated with *M. anisopliae* + 2 untreated *C. smaragdinus*.
- Treatment IV (BioControl 3.0): 10 untreated locusts + 2 *C. smaragdinus* pre-inoculated with *M. anisopliae* (each beetle received 2 μL of a conidial suspension containing 1 × 10^8^ conidia per milliliter (conidia/mL) before release).
- Control: 10 untreated locusts (no fungus, no beetles).

Treatment I involved both beetles and locusts without *M. anisopliae*. Treatment II included the application of *M. anisopliae* to locusts in the absence of *C. smaragdinus*. Treatment III applied *M. anisopliae* to locusts, followed by the addition of two *C. smaragdinus* (1 male and 1 female) to each cage. Treatment IV comprised two *C. smaragdinus* pre-inoculated with *M. anisopliae*, which were then introduced into cages containing 10 locusts. Treatment 5 served as the control, consisting of untreated locusts only.

For all treatments involving *M. anisopliae*, each insect was dosed with a 2 μL solution of *M. anisopliae* (MA2013107391001) (1 × 10^8^/mL) and 0.2% Tween 80. The control group received a 0.2% Tween 80 solution.

Each treatment included 50 third-instar locusts (for all five replicates), due to the potential for cannibalism at high densities, 10 locusts per treatment were introduced into each cage. This resulted in a total of 25 cages (five treatments × 5 replicates/treatment), with five replicates per treatment. Therefore, in treatments involving beetles, there was a 1:5 ratio of beetles to locusts.

Locusts were fed with bouquets of wheat seedlings, and old seedlings were replaced daily with fresh ones. The number of dead locusts and beetles was recorded and collected to assess locust mortality in the presence or absence of *M. anisopliae* and *C. smaragdinus*. Dead *C. smaragdinus* individuals were replaced with new individuals held in separate cages in an insectary. These replacements were starved for 24 h before being introduced into a cage.

Cadavers from the control and *M. anisopliae* treatments were transferred to Petri dishes covered with wetted sterile filter paper (distilled water) and kept at a temperature of 30 °C with a light–dark cycle of 12 h each for seven days. Afterward, the cadavers were examined for the presence of the entomopathogen. Data were collected until all individuals had died. The semi-field experiment was conducted at ES-CPP, SDAU, and lasted for a duration of 20 days, encompassing the mortality of all locusts in Treatments I-IV.

To conceptualize the experimental design, Treatments I and II were considered the traditional approach to biocontrol (BioControl 1.0). Conversely, Treatments III and IV represented the combination of biocontrol agents and were designated as BioControl 2.0 and 3.0. Although both Treatment III and Treatment IV involved the application of two biological agents, the timing of combination differed significantly (Table 1).

### 2.6. Statistical Analyses

The corrected mortality was calculated according to Abbott’s formulas [35] as follows:

(1) $$\mathrm{The}\;\mathrm{corrected}\;\mathrm{mortality}\;=\;(1\;-\;\frac{Nt\;}{Ncol})*100$$

where *Nt* is the total number of locusts after treatment under treated conditions, and *Ncol* is the total number of locusts under the control.

*t*-test and ANOVA were applied to compare the corrected mortality rates among different treatments and different factors. Multiple differences among treatment means were tested using Tukey’s HSD at a significance level of *p* = 0.05. Interaction analyses between predation and infection in III, and IV were analyzed using linear regression and their parameters were summarized as BioControl 2.0 and BioControl 3.0, respectively. Additionally, the Johnson-Neyman procedure was applied corresponding to each interaction model [36] to identify significant interaction regions. All analyses were performed using the R software (version 3.5.1) [37] with “stats” for ANOVA and “interactions” for Johnson–Neyman analyses.

## 3. Results

### 3.1. The Virulence of the M. anisopliae on the Third-Instar of L. migratoria Manilensis

**Figure 1:**
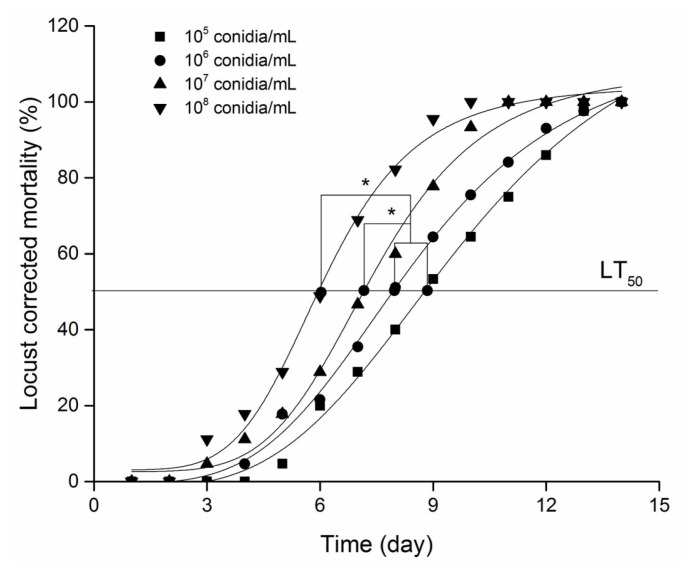
Bioassay of different concentrations of *M. anisopliae* on the third-instar *L. migratoria manilensis*. * indicates the significance of LT_50_/d value at *p* < 0.05 using Tukey’s HSD.

Mortality increased significantly with increasing concentration of *M. anisopliae* (Figure 1), and by the end of the 15th day, all locusts in the five treatments had died. The LT_50_/day (time to 50% mortality) was 9, 8, 7, and 6 days for conidial concentrations of 10^5^ (A), 10^6^ (B), 10^7^ (C), and 10^8^ (D) conidial/mL, respectively. Although there was no significant difference in LT_50_/day between treatments A and B, significant differences were observed between treatments A and C, B and C, as well as D and A, D and B (Figure 1).

### 3.2. Bioassay of M. anisopliae Against C. smaragdinus

**Figure 2:**
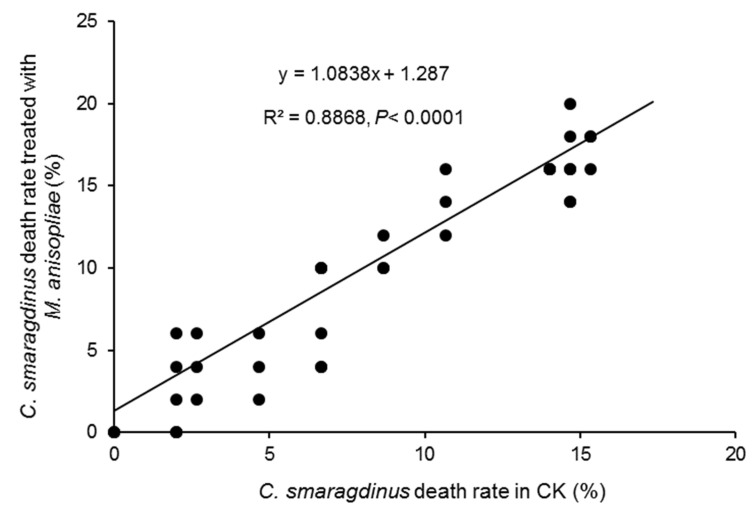
Linear relationship between death rates for control and *M. anisopliae* treatments.

In the bioassay of *M. anisopliae* against *C. smaragdinus*, we monitored the mortality and muscardine cadaver rate of *C. smaragdinus* in different treatments every three days. After 36 days, *C. smaragdinus* exhibited similar viability in both the control and *M. anisopliae* treatments shown by their mortality. The mortality rate of *C. smaragdinus* treated with *M. anisopliae* was not statistically significant compared to the control but was close to the threshold for significance (*t*-test, df = 12, *t* = −2.15, *p* = 0.0528), which did not reach the conventional significance level of *p* < 0.05. The mortality rate of *M. anisopliae* correlated with the mortality rate of the control treatment (y = 1.0838x + 1.287, *R*^2^ = 0.8868, *p* < 0.0001), with a correlation coefficient of 0.97 (*p* < 0.0001), suggesting that *M. anisopliae* does not have a significant effect on *C. smaragdinus* mortality (Figure 2). Additionally, no muscardine was observed on cadavers preserved for 10 days at RH = 80% and 28 °C.

### 3.3. Assessing the Combined Effect of C. smaragdinus and M. anisopliae on the Efficacy of L. migratoria Control

**Figure 3:**
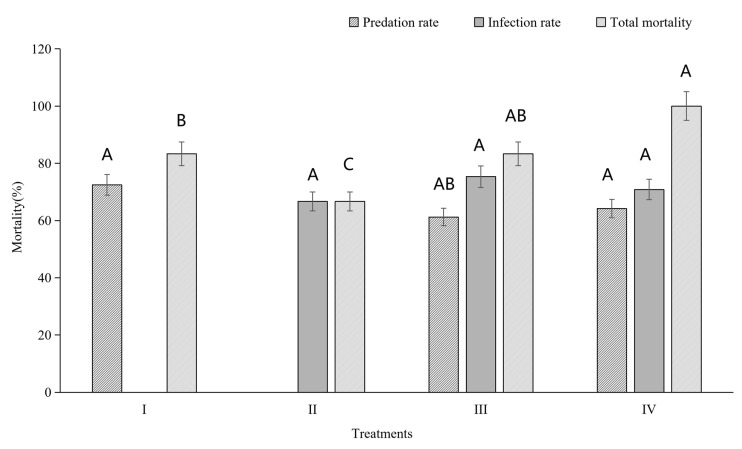
Predation, fungal infection, and total corrected mortality of *Locusta migratoria manilensis* under different biocontrol treatments. Treatments include predator only (I), fungus only (II), sequential combination (BioControl 2.0; III), and predator-mediated delivery (BioControl 3.0; IV). Data are corrected for control mortality. Bars represent mean ± SE. Different letters indicate significant differences among treatments (Tukey’s HSD, *p* < 0.05).

In the field cage experiment, four treatments involving two different biological systems were conducted. The total locust mortality rate in Treatment IV was significantly higher than in Treatments I and II (Figure 3). However, the total locust mortality rate in Treatment III was only significantly higher than in Treatment II, not Treatment I (Figure 3). No significant differences were observed in predation rates or infection rates among the different treatments (Figure 3). Interaction analyses revealed that predation had a significant effect on locust mortality, and there was a significant negative interaction between predation and infection in BioControl 2.0 (Treatment III) (Table 2 and Table 3). However, there was no significant interaction between predation and infection in BioControl 3.0 (Treatment IV, Table 2 and Table 3). In contrast, both predation and infection significantly caused locust death BioControl 3.0 (Table 2 and Table 3).

### 3.4. Synergistic Effect of Predation and Infection on L. migratoria in BioControl 3.0

**Figure 4:**
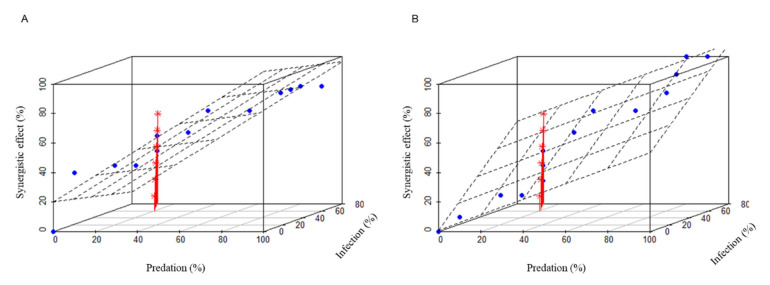
The synergistic effect of predation and infection on locusts in Treatment 3 (**A**) and Treatment 4 (**B**). Red colored lines are plotted with an asterisk as their point symbol, which indicate the synergistic effects of predation and infection. The blue dots indicate the samples with different predation and infection.

A significant increase in mortality rate was observed in treatments BioControl 2.0 (Treatment III) and BioControl 3.0 (Treatment IV) compared to conventional methods (Treatment I and II, BioControl 1.0) (Figure 3). To understand how these two biological agents interact and work together on locusts, we further investigated their synergistic effect. Our analyses indicate that distinct mechanisms underlie seemingly similar combinations in two different systems for controlling locusts. In Treatment III, overall mortality increased within the standard deviation of predation when infection was the moderator. However, the response of overall mortality to increases in infection varied when predation was the moderator. In Treatment IV, regardless of whether the moderator was infection or predation, overall mortality increased in parallel with the increase in either predation or infection within the range of their standard deviation, indicating that predation and infection acted independently on locusts (Figure 4).

As indicated in Table 2, infection and predation exhibited a negative interaction on locust mortality in both Treatment III (BioControl 2.0) and Treatment IV (BioControl 3.0). However, this interaction did not impact the mortality of locusts in Treatment IV (BioControl 3.0). It is noteworthy that both predation and infection significantly affected locust mortality in Treatment IV (Table 3, BioControl 3.0). However, in Treatment III (Table 3, BioControl 2.0), predation, as well as the interaction between predation and infection, but not infection alone, had a significant impact on locust mortality. This suggests that slight differences in setting up the biological complex can result in distinct outcomes (Table 2 and Table 3).

The Johnson-Neyman plots provide further insights into the underlying mechanism of predation-infection interaction. For BioControl 2.0, infection significantly affected the slope of predation (*p* < 0.01) when mortality caused by infection was less than 39.44. Predation, on the other hand, did not impact the slope of infection (*p* < 0.01) within the range of [0, 1]. In BioControl 3.0, infection did not influence the slope of predation, while predation affected the slope of infection (*p* < 0.01) when mortality caused by predation was within the range of [−71.61, 36.32]. It is worth noting that, regardless of the significant effects of one factor on the other, both infection and predation exhibited an antagonistic (negative) effect on each other. However, this antagonistic effect was larger and significant in Treatment III (BioControl 2.0), while it was not significant in Treatment IV (BioControl 3.0).

## 4. Discussion

Locusts are among the world’s most destructive pests that cause significant financial loss and ecological damage in many parts of the world. It is noteworthy that, in China, locust outbreaks have a 3000-year history, and along with floods and droughts, are considered the three biggest natural disasters for the country [38]. However, locust control typically relies largely on chemical pesticides due to their fast action despite more negative impacts on the environment, increasing the risks to humans and wildlife. Biological agents have great potential, but their uptake has been hampered by inconsistent efficacy, costs, and limited knowledge of application techniques. Traditionally, single BCAs were used repeatedly (the so-called “BioControl 1.0” approach), but efforts have shifted toward combining complementary agents (“BioControl 2.0”) to improve outcomes. For example, combining *M. anisopliae* with a locust-pathogenic bacterium *P. pseudoaligenes* has shown enhanced suppression [17]. However, our understanding of multi-agent interactions is still evolving: recent analyses suggest that true synergistic interactions are rare and that antagonism between agents is more common [39].

In this study, we demonstrate BioControl 3.0, which harnesses a predator to vector an entomopathogen. This biocontrol complex combines pest pathogens and natural predatory enemies that not only incorporates infection and predation to exert direct biotic pressure on pest populations but also facilitates microbial delivery, thereby achieving a triple mode of action. We showcase the BioControl 3.0 is more effective than 2.0 and 1.0 using a case study of *Locusta migratoria* control. Specifically, this complex controls locusts by transmitting *M. anisopliae* using *C. smaragdinus* as the mediator. In BioControl 3.0, where beetles vectored the fungus, presented an additive effect with predation and infection, achieving the best results. In contrast, BioControl 2.0 (Treatment III) showed a negative interaction, likely because infected locusts were less appealing to beetles, reducing predation. This study confirms that the *C. smaragdinus* and *M. anisopliae* complex, especially when beetles carry the fungus, significantly enhances locust control beyond single-agent use.

The efficacy of such biocontrol complexes depends on several factors. First, the agents must have complementary roles and minimal antagonism. Both *M. anisopliae* and *C. smaragdinus* were individually effective against locusts [32], but as we saw, not all combinations perform better. Second, understanding the interactions between different BCAs within the complex is vital. Synergistic interactions, where the combined effect of multiple BCAs is greater than the sum of their individual effects, can significantly enhance biocontrol efficacy. Conversely, antagonistic interactions, such as competition or predation between BCAs, can reduce their effectiveness. Compared to our previous study, which introduced this combination, this work provides deeper analysis of interaction dynamics and application strategies, and showed that simply combining biological agents may result in a negative interaction between agents, whereas in BioControl 3.0 (simultaneous release with vectoring) both agents acted in parallel, enhancing the control efficacy. Third, deployment configuration governs interaction dynamics and efficacy. In BioControl 2.0 (Treatment III), sequential deployment of *M. anisopliae* followed by *C. smaragdinus* produced a significant yet negatively interacting effect: infection initially drove mortality up to a threshold (39.44% mortality), beyond which predation impact on mortality diminished (Table 2). Conversely, predation did not significantly modulate infection’s effectiveness across its observed range. This antagonism curtailed overall efficacy, as only predation, and not infection alone, had a significant effect on locust death (Table 3). In BioControl 3.0 (Treatment IV), simultaneous application decoupled their effects: both infection and predation independently and significantly increased mortality (Table 3), with no significant negative interaction (Table 2). Here, predation modestly influenced infection-driven mortality when predation-caused mortality lay between 36.32% and 100%, but this did not translate into a measurable antagonistic reduction in total efficacy. These contrasting outcomes, arising solely from differences in timing and sequence, underscore that even identical agent combinations can yield divergent control performance. Optimizing BioControl 3.0 thus requires not only selecting complementary agents but also fine-tuning their application schedule to mitigate antagonism and maximize independent or synergistic mortality effects.

Pathogen transmission dynamics are also pivotal in determining the long-term sustainability of biological control. In our system, *M. anisopliae* can disperse via multiple pathways: direct contact between infected and susceptible locusts, environmental vectors (wind and rain), and, crucially, biological mediators such as *C. smaragdinus* and plant surfaces. *C. smaragdinus* possesses strong dispersal capabilities and is known to travel considerable distances across heterogeneous landscapes [24,27], making it an ideal vehicle for disseminating fungal spores. This mediator-assisted dispersal not only enhances immediate control but may also facilitate landscape-level persistence of the pathogen.

**Figure 5:**
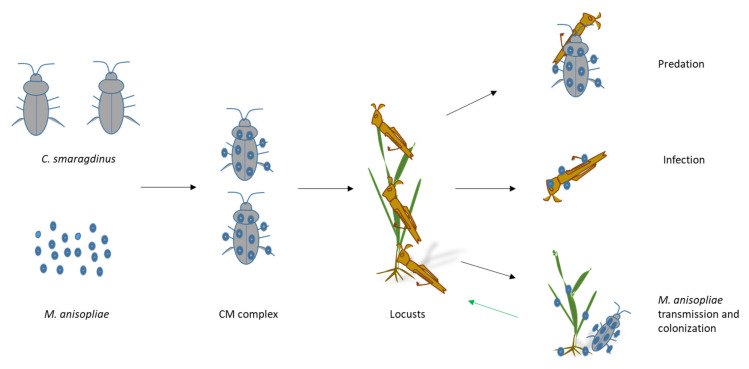
The flow chart and mechanism of biocontrol complex.

Beyond acute pest suppression, *M. anisopliae* may contribute to longer-term ecological benefits. Studies have shown that this fungus can colonize plant tissues endophytically, promoting growth and stress resistance [40,41,42]. Additionally, it suppresses locust aggregation behavior [43], potentially limiting outbreak initiation. Therefore, in the BioControl 3.0 framework (Figure 5), *M. anisopliae* serves dual roles: pest suppression and plant growth promotion—offering a self-reinforcing, ecologically embedded pest management system. The capacity for long-term endophytic residency further enhances the sustainability of this approach.

## 5. Conclusions

We present BioControl 3.0 as a synergistic pest management strategy that leverages predator–pathogen interactions. Our BioControl 3.0 framework achieves a three-pronged mechanism: direct predation, pathogen-mediated mortality, and beetle-mediated pathogen dissemination. Our results confirm that combining *M. anisopliae* and *C. smaragdinus* in a vectoring configuration significantly enhances locust mortality over single-agent treatments. The outcomes highlight that agent compatibility and deployment strategy are key: simply mixing BCAs can backfire, but strategic use of a predator as a fungal carrier achieves additive (non-antagonistic) control. This work provides a proof-of-concept for integrated biological control complexes and offers guidance for developing ecologically based, dual-agent systems for locust management. 

At the same time, the present conclusions are drawn from field-cage experiments designed to resolve mechanistic interactions under controlled conditions. The performance, ecological safety, and operational feasibility of BioControl 3.0 under open-field and landscape-scale scenarios remain to be evaluated. Future research should therefore focus on validating efficacy under realistic environmental conditions, assessing long-term ecological effects, and optimizing deployment strategies within integrated pest management frameworks. Such efforts will be essential for translating this conceptual advance into a practical and sustainable tool for locust control.

Beyond locust control, the predator-vectored pathogen strategy proposed here may be applicable to other insect pests that exhibit gregarious behavior, high mobility, or frequent contact with ground-dwelling natural enemies. Potential targets include other Orthopteran pests such as grasshoppers and crickets, as well as soil- and ground-associated agricultural pests including cutworms (*Agrotis* spp.), wireworms (*Elateridae larvae*), and certain coleopteran larvae. In these systems, native predatory beetles or other generalist predators could potentially serve as vectors for entomopathogenic fungi or other microbial agents. In addition, predator–pathogen complexes may complement existing biological control programs in orchard and forest ecosystems, provided that agent compatibility, non-target effects, and ecological context are carefully evaluated. Together, these considerations suggest that BioControl 3.0 represents a broadly transferable concept for developing integrated, ecologically based pest management strategies across diverse agroecosystems.

## Figures and Tables

**Table 1:** Details of different treatments used in field experiments.

Treatments	Treatment Detail
Control	Locusts only (no beetles, no fungus)
I (BioControl 1.0—predator)	Locusts with untreated *C. smaragdinus*
II (BioControl 1.0—fungus)	Locusts treated with *M. anisopliae*
III (BioControl 2.0)	*M. anisopliae*-treated locusts with untreated *C. smaragdinus*
IV (BioControl 3.0)	Locusts with *M. anisopliae*-inoculated *C. smaragdinus*

**Table 2:** Interaction analyses of *M. anisopliae* and *C. smaragdinus* on *L. migratoria* mortality.

	Factors	Estimate	Std. Error	*t* Value	*p* (>|t|)
BioControl 2.0	(Intercept)	13.4323	4.8612	2.763	0.01386 *
	Predation	0.9104	0.1808	5.034	0.0001 ***
	Infection	0.4870	0.3317	1.468	0.1614
	Predation: Infection	−0.0073	0.003219	−2.259	0.0382 *
	Whole model	*R*^2^ = 0.9029	6.773	59.88	6.433 × 10^−9^ ***
BioControl 3.0	(Intercept)	−0.9287	5.0883	−0.183	0.8575
	Predation	0.5329	0.1892	2.815	0.0124 *
	Infection	0.8579	0.3472	2.471	0.0251 *
	Predation: Infection	−0.0019	0.0034	−0.577	0.5722
	Whole model	*R*^2^ = 0.9577	7.089	144.4	8.502 × 10^−12^ ***

Statistical significance: ‘***’ 0.001 ‘*’ 0.05. BioControl 2.0 indicates the treatment III, and BioControl 3.0 indicates the treatment IV in Table 1.

**Table 3:** Analysis of variance factors in Models 1 and 2.

	Factors	df	Sum Sq	*F* Value	*p* (>F)
BioControl 2.0	Predation	1	7974.2	173.8243	5.202 × 10^−10^ ***
	Infection	1	32.0	0.7151	0.4102
	Predation: Infection	1	234.0	5.1011	0.03823 *
	Residuals	16	734.0		
BioControl 3.0	Predation	1	21,055.3	418.9261	6.698 × 10^−13^ ***
	Infection	1	703.8	14.0029	0.0018 **
	Predation: Infection	1	16.7	0.3326	0.5721
	Residuals	16	804.2		

Statistical significance: ‘***’ 0.001 ‘**’ 0.01 ‘*’ 0.05. BioControl 2.0 indicates the treatment III, and BioControl 3.0 indicates the treatment IV in Table 1.

## Data Availability

The datasets used and/or analyzed during the current study are available from the corresponding author on reasonable request.
